# Injectable agarose hydrogels and doxorubicin-encapsulated iron-gallic acid nanoparticles for chemodynamic-photothermal synergistic therapy against osteosarcoma

**DOI:** 10.3389/fchem.2022.1045612

**Published:** 2022-11-01

**Authors:** Hongliang Ying, Haitian Wang, Guangchuan Jiang, Han Tang, Lingrui Li, Jinrui Zhang

**Affiliations:** ^1^ Department of Orthopedics, China-Japan Union Hospital of Jilin University, Changchun, China; ^2^ Key Laboratory of Artificial Micro- and Nano-Structures of Ministry of Education, School of Physics and Technology, Wuhan University, Wuhan, China; ^3^ College of Medicine, Zhengzhou University, Zhengzhou, China

**Keywords:** iron-gallic acid, chemodynamic therapy, photothermal therapy, self-sufficient H_2_O_2_, agarose hydrogel

## Abstract

Osteosarcoma is a malignant bone cancer that usually occurs in children and adolescents. Although chemotherapy, radiotherapy and other methods have been used to treat osteosarcoma, these therapeutic regimens fail to cure this disease completely. Herein, doxorubicin-encapsulated iron–gallic acid (FeGA-DOX) nanoparticles (NPs) were fused with agarose hydrogels (AG) for synergistic therapy of osteosarcoma. Under near-infrared laser irradiation, the local temperature of FeGA-DOX NPs was increased. Therefore, tumour cells were killed using photothermal therapy, and AG dissolved to release FeGA-DOX into the cells. Doxorubicin generates hydrogen peroxide, which is then converted to reactive oxygen species (ROS) *via* FeGA-DOX by the Fenton reaction, inducing tumour cell apoptosis. ROS induced by chemodynamic therapy compensates for the incomplete cure of osteosarcoma cells. The AG-encapsulated NPs could mediate synergistic chemodynamic and photothermal therapy with self-sufficient H_2_O_2_, providing a novel therapeutic strategy for osteosarcoma.

## Introduction

Osteosarcoma is one of the most common cancers in children and adolescents that mainly occurs in the metaphyseal region of long bones, especially the limbs and shoulders, and often leads to metastasis (Ottaviani and Jaffe 2010; Czarnecka et al., 2020). The clinical symptoms of osteosarcoma include swelling, severe pain and joint movement limitation (Lopes-Júnior et al., 2018; Hameed, Horvai, and Jordan 2020). Multi-therapeutic methods have been utilised to treat osteosarcoma, including chemotherapy, radiotherapy, surgery, targeted therapies and other regimens (Gill and Gorlick 2021). However, recurrence occurs during treatment because of an incomplete cure (Meltzer and Helman 2021). Therefore, much research has tried to take the advantage of nanoparticles (NPs) to develop effective novel therapeutic methods for the treatment of osteosarcoma (Lu et al., 2018; Du et al., 2021).

The tumour microenvironment is characterised by acidity, high H_2_O_2_ and glutathione levels (H. Chen et al., 2022; D. Zhu, et al., 2022; Lyu et al., 2021a). Chemodynamic therapy (CDT) involves utilising transition metal-containing NPs to generate *in situ* cell toxic reactive oxygen species (ROS) in cancer cells from this intracellular overexpressed H_2_O_2_ (Tian et al., 2021; X. Wang et al., 2020; M. Lyu, et al., 2020b; Q. Chen et al., 2019; Ranji-Burachaloo et al., 2018; M. Lyu et al.). This chemical process is called “Fenton reaction” or “Fenton-like reaction” (Ranji-Burachaloo et al., 2018; Meng et al., 2020). The produced ROS respond to the unique tumour microenvironment and can induce cancer cell apoptosis with high cell toxicity (Yan et al., 2021). For example, copper-based nanoplatforms, such as Cu–cys, react with this intracellular H_2_O_2_ to produce hydroxyl radicals (Ma et al., 2019; M. Wang et al., 2022). However, it is well known that the Fe-induced Fenton reaction is efficient in strongly acidic conditions, and the reaction efficiency is highly related to acidity conditions. Additionally, because H_2_O_2_ is overexpressed in tumour tissues compared with normal tissues, the amount of H_2_O_2_ is still inadequate if taken as the only source of ROS production to cure cancer and prevent metastasis or recurrence (Dong et al., 2020; X. Lyu, et al., 2021).

Photothermal therapy (PTT), with the assistance of NPs, has emerged as one of the novel treatment methods for cancers (M. Lyu et al., 2021). Upon laser irradiation, NPs, named PTT agents, absorb the energy of light and convert it to heat for local hyperthermia therapy. Near-infrared (NIR) wavelength ranging from 650 to 1,800 nm is transparent to the biological tissues, and nano-agents with NIR window could trigger PTT in deep tumours (Hong, Antaris, and Dai 2017; Pascal et al., 2021). These nano-agents can be grouped into inorganic and organic materials. Inorganic materials include novel materials such as gold (Aioub, Panikkanvalappil, and El-Sayed 2017), palladium (Zhang et al., 2018), carbon-based materials such as carbon tubes (Liang et al., 2014) and C60 (Hu et al., 2017). Molecules and some polymer NPs were categorised as organic materials (Liu et al., 2020). However, the penetration depth of PTT is relatively limited, and therefore the therapeutic effect sometimes fails to meet the clinical requirement (Duo et al., 2022). Therefore, it is essential to enhance the treatment outcome of PTT by combining PTT with other therapeutic regimens.

**SCHEME 1:**
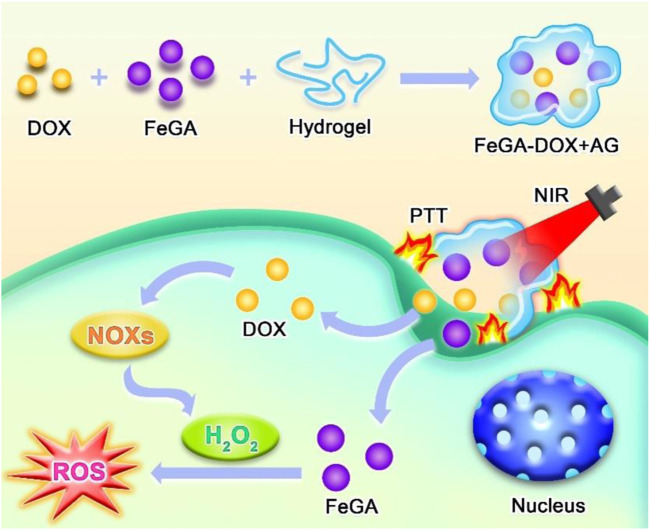
Illustration of synthesis of FeGA-DOX + AG and the intracellular mechanism of FeGA-DOX + AG under CDT/PTT.

Gallic acid (GA) is a natural polyphenolic compound that is present in rhubarb, eucalyptus, dogwood and other plants (You et al., 2010; Park 2017). Additionally, it possesses remarkable biocompatibility and can be easily absorbed by the human body. GA has been widely applied for antitumour or anticancer treatments (Zeng et al., 2016). Iron element was widely applied in tumour therapy (Cheng et al., 2021; Y. Zhu et al., 2021; Y. Zhu et al., 2020; Y. Zhu et al., 2022; W. Wang et al., 2021). Herein, we first synthesised uniform iron–GA (FeGA) and constructed an agarose hydrogel (AG)-encapsulated iron–GA decorated with doxorubicin (FeGA-DOX) nanoplatforms for the treatment of osteosarcoma. FeGA-DOX and AG were injected into the tumour in mice. The mechanism of tumour cell death induced by FeGA-DOX + AG is shown in Scheme 1. At irradiation of the NIR laser, the FeGA-DOX NPs absorb the laser’s energy and induce hyperthermia leading to PTT in tumour cells. Meanwhile, the AG was melted under heating, followed by the release of encapsulated FeGA-DOX. The released doxorubicin activates nicotinamide adenine dinucleotide phosphate (NADPH) oxidases, producing abundant H_2_O_2_. Together with overexpressed H_2_O_2_ in tumor site, FeGA reacts with the produced H_2_O_2_ to generate abundant ROS under a mild acidic tumour microenvironment for performing CDT by the Fenton reaction, which induced tumour cell death. By this method, the growth of osteosarcoma tumours in mice could be efficiently suppressed without severe side effects. This study presents a novel synergistic CDT and PTT strategy for osteosarcoma treatment.

## Results and discussion

**FIGURE 1:**
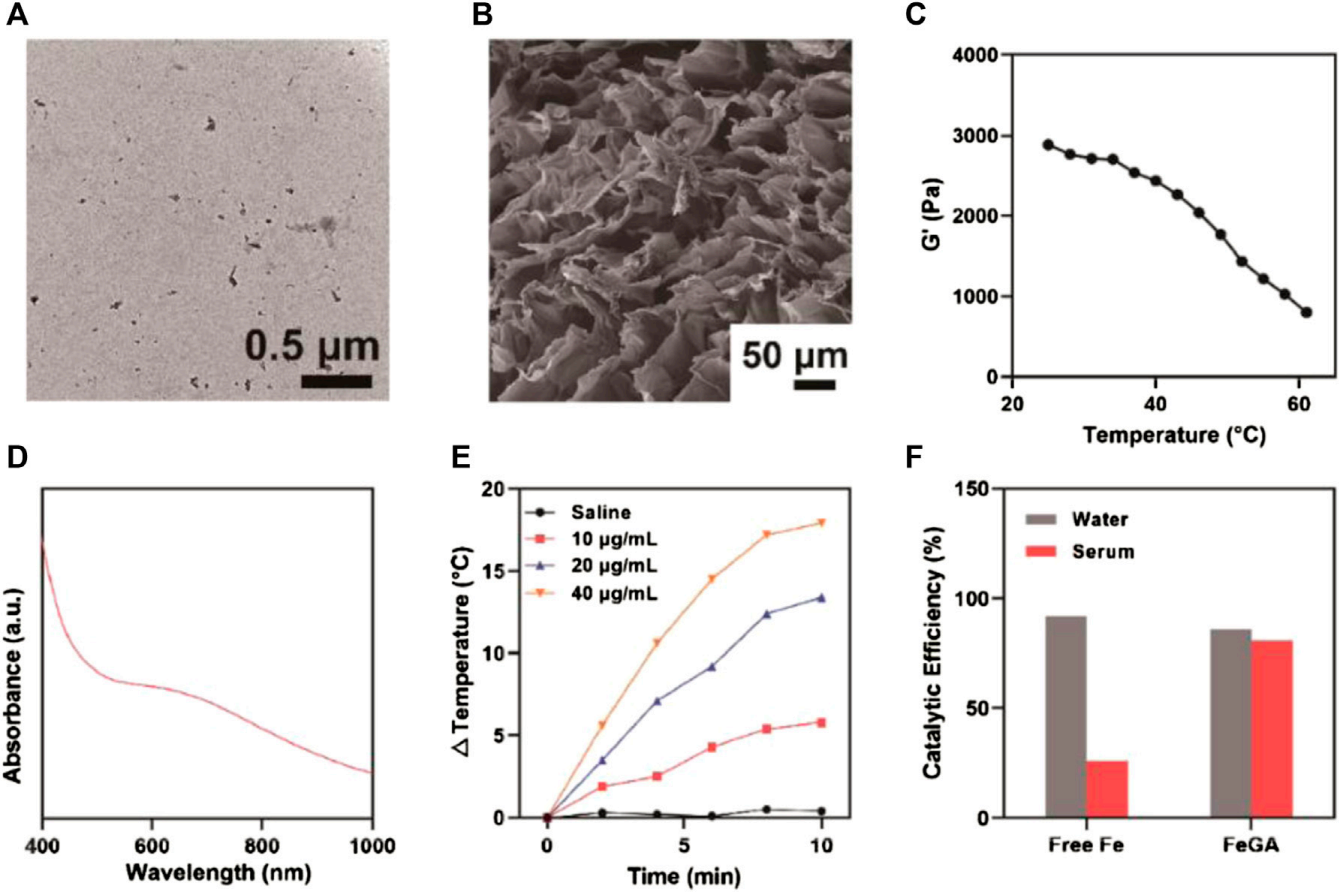
**(A)** Transmission electron microscopy image of FeGA-DOX. **(B)** SEM image of FeGA-DOX + AG. **(C)** Rheological behaviour analysis of FeGA-DOX + AG. **(D)** UV–vis absorption of FeGA-DOX. **(E)** The photothermal heating curve of FeGA-DOX at various concentrations. **(F)** The relative catalytic activity of free Fe^2+^ and FeGA-DOX in water and serum.

The prepared FeGA-DOX + AG NPs with controlled release in thermal response are shown in Scheme 1. We used the strong coordination between GA polyphenol groups and ferrous ions (Fe(II)) to prepare ultra-small metal polyphenol network FeGA nanocomplexes, which were then encapsulated in hydrogels with the chemotherapeutic drug DOX. Under transmission electron microscopy, the prepared FeGA-DOX nanocomplexes had uniform size distribution, with an average diameter of 2.5 nm (Figure 1A). Scanning electron microscope (SEM) image showed a complex pore structure of the hydrogel (Figure 1B). Subsequently, we measured the rheological values of FeGA-DOX + AG at different temperatures (Figure 1C). The rheological behaviour analysis results showed that with an increase in temperature, FeGA-DOX + AG gradually dissolved and the storage modulus gradually decreased, which was consistent with the characteristics of hydrogels. Figure 1D depicts the absorption of FeGA-DOX. FeGA-DOX has a broad absorption spectrum of 600–800 nm, indicating that FeGA-DOX has a high potential for photothermal conversion in the NIR-I region. Therefore, dispersions of FeGA-DOX with different concentrations were irradiated with an 808 nm laser for 10 min, and the photothermal heating curve was drawn to study its photothermal conversion performance (Figure 1E). It should be pointed out that after several heating-up and cooling-down loop, FeGA-DOX maintained its photothermal stability (Supplementary Figure S1). Most importantly, Figure 1F shows that compared with the catalytic activity in the water, the Fenton catalytic activity of free Fe^2+^ ions will be significantly inhibited in serum. In contrast, FeGA-DOX has excellent and stable Fenton-like catalytic activity in water and serum. Therefore, FeGA-DOX with good catalytic activity and physiological environment stability has broad biological application prospects.

**FIGURE 2:**
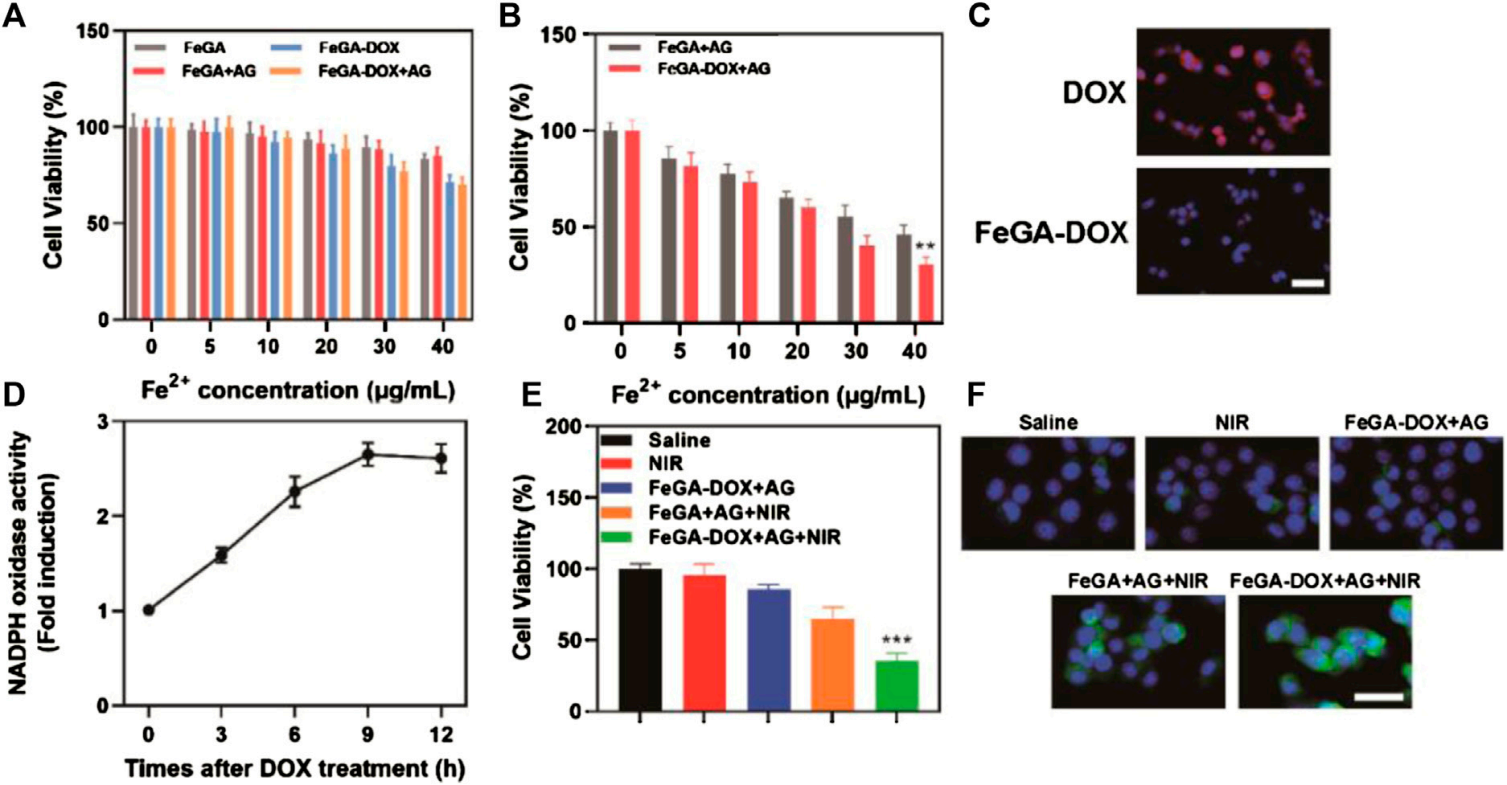
**(A)** Cytotoxicity of FeGA, FeGA-DOX, FeGA + AG and FeGA-DOX + AG on the K7M2wt cells. **(B)** Cell viability of FeGA + AG and FeGA-DOX + AG on the K7M2wt cells under laser irradiation. **(C)** CLSM images of intracellular H_2_O_2_ levels in the K7M2wt cells incubated with DOX and FeGA-DOX (scale bar: 50 μm). **(D)** Nicotinamide adenine dinucleotide phosphate (NADPH) oxidase activity measurements after co-incubation with FeGA-DOX + AG. **(E)** Cell viability of the K7M2wt cells after various treatments. **(F)** CLSM of ROS generation in various treatment groups (scale bar: 50 μm).

The K7M2wt cells were incubated with FeGA, FeGA-DOX, FeGA + AG and FeGA-DOX + AG to explore their dark toxicity. Even if the dose of FeGA-DOX + AG reached 40 μg/ml, the cell survival rate was 70.1% and no apparent cytotoxicity was found (Figure 2A). Subsequently, the cell viability of FeGA + AG and FeGA-DOX + AG was detected using the cell counting kit eight method in the presence of NIR laser irradiation to verify the ability of FeGA-DOX + AG *in vitro* photothermal-enhanced CDT. As shown in Figure 2B, after NIR laser irradiation with FeGA-DOX + AG at a dose of 40 μg/ml, the cell survival rate was reduced to 30.6%, which was reduced by half compared with that of the group without laser irradiation in Figure 2A. Subsequently, we used fluorescence microscopy to assess the intracellular H_2_O_2_ levels in the K7M2wt cells, according to confocal laser scanning microscope images (Figure 2C). It can be seen that red fluorescence is uniformly distributed in the cells in the DOX group, indicating that DOX can produce a large amount of H_2_O_2_ in the cells. The determination results of NADPH oxidase activity (Figure 2D) also suggested that DOX had NADPH oxidase activity, which could consume NADPH molecules to form superoxide radicals, producing H_2_O_2_. The cell survival rate of each group in Figure 2E was also consistent with that of our previous studies, which verified that FeGA-DOX + AG could provide synergistic photothermal enhancement of CDT *in vitro*. However, the red fluorescence intensity of the FeGA-DOX group was significantly reduced, indicating that the increase in intracellular H_2_O_2_ levels was conducive to the redox reaction with FeGA to generate ROS. Therefore, we proposed that the ROS level of the FeGA-DOX group would be higher than that of the saline group, and the FeGA-DOX + AG group in Figure 2F verified our assumption. Under laser irradiation, the ROS fluorescence intensity of the FeGA-DOX + AG + NIR group was significantly increased.

**FIGURE 3:**
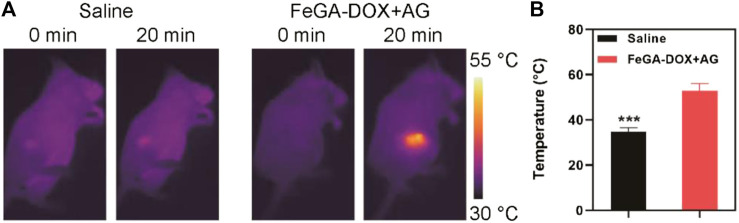
**(A)** IR images of mice after treatments and **(B)** temperature at 20 min post-irradiation. *p* values in **(B)** were calculated by Tukey’s post-test (***p < 0.001).

Given the excellent performance of FeGA-DOX + AG as nano-agent *in vitro*, we used Balb/c mice to establish a K7M2wt subcutaneous tumour model to continue to study its effect on photothermal conversion *in vivo* (Figures 3A,B). It is not difficult to understand from Figure 3B that with the increase in irradiation time, the temperature in the tumour site of the mice in the FeGA-DOX + AG group increased to 52.9°C. In comparison, the temperature in the tumour site of mice in the control group only increased by 2.8°C, which can be seen from the infrared radiation images of mice (Figure 3A), indicating that FeGA-DOX + AG has superior photothermal conversion capacity *in vivo*.

**FIGURE 4:**
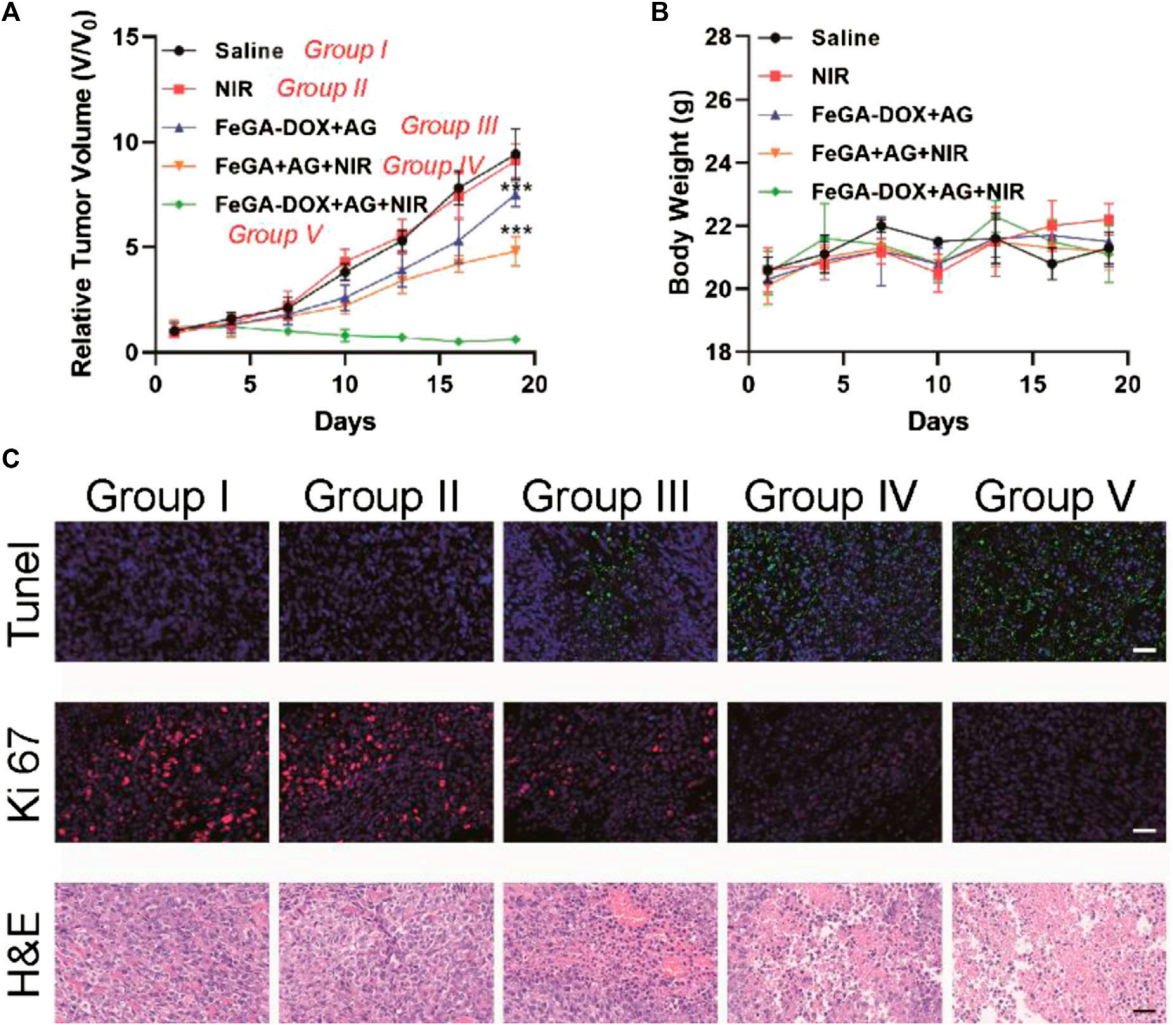
**(A)** Tumour volume and **(B)** body weight of K7M2wt tumour-bearing mice in the saline, NIR, FeGA-DOX + AG, FeGA + AG + NIR and FeGA-DOX + AG + NIR groups. **(C)** Representative images of TUNEL, Ki-67 and H&E antibody staining in all treatment groups (Scale bar: 50 μm). *p* values in **(A)** were calculated by Tukey’s post-test (***p < 0.001).

We tested FeGA-DOX + AG-mediated antitumour activity in the K7M2wt tumour-bearing mice. A tumour model was established by subcutaneously injecting 1 × 10^6^ K7M2wt cells into the right leg of Balb/c mice. When the volume of the primary tumour reached 200 mm^3^, the mice were randomly divided into five groups (five mice in each group): Group I: saline; Group II: NIR; Group III: FeGA-DOX + AG; Group IV: FeGA + AG + NIR and Group V: FeGA-DOX + AG + NIR. Tumour volume increased rapidly in the saline and NIR treatment groups within nearly 2 weeks of treatment, as shown in Figure 4A. Group III has little tumour suppressive effect, whereas the tumour suppressive effect of Group IV is almost moderate. This is because, after laser irradiation, FeGA is released from the hydrogel, which is more likely to react with H_2_O_2_ in the tumour microenvironment to generate ROS to destroy mitochondria, thus improving the chemotherapy sensitivity and tumour killing ability. Hence, in the most effective treatment group, the growth curve of tumour volume in Group V was almost completely inhibited during treatment. Most importantly, no significant changes in the body weight of the treated mice were observed in five groups, which suggest no significant systemic toxicity in all treatment methods (Figure 4B). This result exhibited promising potential for future medical applications of this nanomaterial. Finally, the tumour tissues in all groups were sectioned, follows by being stained. The hematoxylin-eosin (H&E) staining results (Figure 4C) showed severe histological damage in tumour sections of Groups IV and V, especially in Group V, with significant cell necrosis. In contrast, no apparent damage was observed in tumour sections of the Group I, Group II and Group III, consistent with the tumour growth curve and TdT-mediated dUTP nick end labelling (TUNEL) apoptosis evaluation results. Ki-67 staining showed that the proliferation of tumour cells in Group IV was significantly inhibited. In contrast, the proliferation of tumour cells in Group V was inhibited entirely, which further proved that our FeGA-DOX + AG had an excellent therapeutic effect.

**FIGURE 5:**
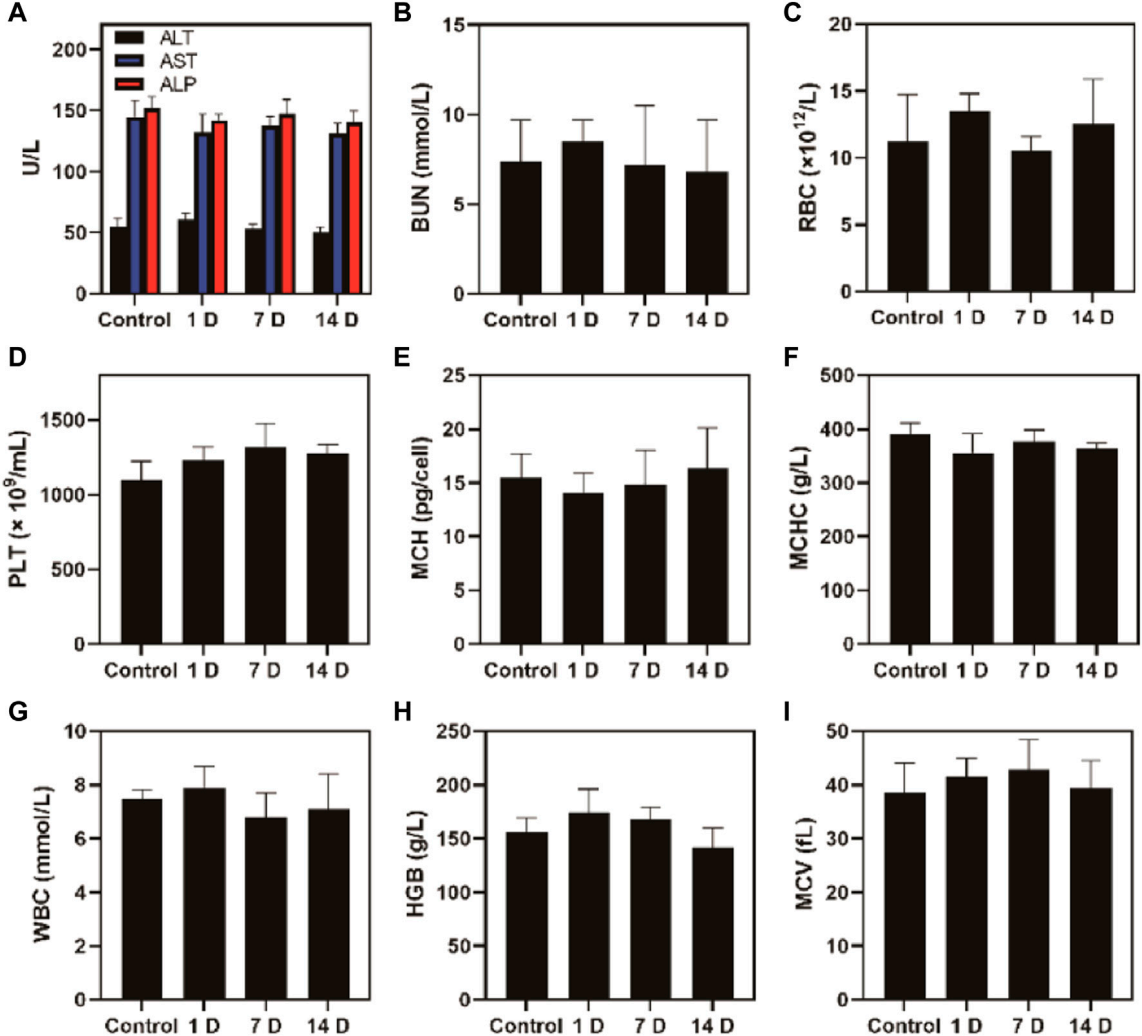
Blood biochemistry analysis of healthy Balb/c mice and mice administrated with FeGA-DOX on days 1, 7 and 14. **(A)** Aspartate aminotransferase (AST), alanine aminotransferase (ALT) and alkaline phosphatase (ALP). **(B)** Blood urea nitrogen (BUN); **(C)** red blood cell (RBC); **(D)** platelets (PLT); **(E)** haemoglobin (HGB); **(F)** mean corpuscular haemoglobin concentration (MCHC); **(G)** white blood cell (WBC); **(H)** mean corpuscular haemoglobin (MCH); **(I)** mean corpuscular volume (MCV).

Additionally, the FeGA-DOX treatment did not cause systemic toxicity risk and system damage in mice, as shown in Figure 5. After treatment, the liver, kidney and blood routine indexes of mice were normal. *In vivo* experiments showed that our unique combination therapy achieved high biosafety, increased the ROS content in tumours and enhanced the antitumour activity.

## Conclusion

In this study, we developed a novel strategy using injectable AG for performing local CDT/PTT with FeGA-DOX NPs to enable osteosarcoma tumour suppression in mice. Upon NIR laser irradiation, the FeGA-DOX NPs emit much heat to induce cell apoptosis by hyperthermia. Meanwhile, a local temperature rise could promote the intratumoural release of FeGA-DOX. It is well known that doxorubicin can promote the generation of H_2_O_2_, which can be converted to ROS *via* a Fenton reaction under acidic conditions by FeGA. Thus, the synergistic effect of CDT/PTT was realised with the assistance of FeGA-DOX + AG treatment. The strategy overcame the limitation of single CDT or PTT and showed outstanding therapeutic outcomes on osteosarcoma tumour-bearing mice with satisfactory biocompatibility results. Hence, this novel approach can integrate the advantage of CDT/PTT by this H_2_O_2_ self-sufficient AG-encapsulated FeGA-DOX, exhibiting potential in clinical applications.

## Data Availability

The original contributions presented in the study are included in the article/Supplementary Material, further inquiries can be directed to the corresponding author.
