# Supplementary material for: Injectable agarose hydrogels and doxorubicin-encapsulated iron-gallic acid nanoparticles for chemodynamic-photothermal synergistic therapy against osteosarcoma
Source: Front Chem. 2022 Nov 1;10:1045612. doi: 10.3389/fchem.2022.1045612 (PMC9663816; doi:10.3389/fchem.2022.1045612)
Supplement: Supplementary file 1 [file DataSheet1.docx]

Supplementary information

**Injectable agarose hydrogels and doxorubicin-encapsulated iron-gallic acid nanoparticles for chemodynamic-photothermal synergistic therapy against osteosarcoma**

Hongliang Ying^1^, Haitian Wang^1^, Guangchuan Jiang^1^, Han Tang^2^, Lingrui Li^3^, Jinrui Zhang*^1^

^1^ Department of Orthopedics, China-Japan Union Hospital of Jilin University, Changchun, 130033, People’s Republic of China

^2^ Key Laboratory of Artificial Micro- and Nano-Structures of Ministry of Education, School of Physics and Technology, Wuhan University, Wuhan, 430072, China

^3^ College of Medicine, Zhengzhou University, Zhengzhou, 450001, Chin

E-mail:

Hongliang Ying: [yinghl@jlu.edu.cn](mailto:yinghl@jlu.edu.cn)

Haitian Wang: [670041956@qq.com](mailto:670041956@qq.com)

Guangchuan Jiang: [jiangguangchuanjd@163.com](mailto:jiangguangchuanjd@163.com)

Han Tang: [2016302180221@whu.edu.cn](mailto:2016302180221@whu.edu.cn)

Lingrui Li: llrdiels@gmail.com

Jinrui Zhang: jlzhangjr@jlu.edu.cn

*Corresponding Author: Jinrui Zhang


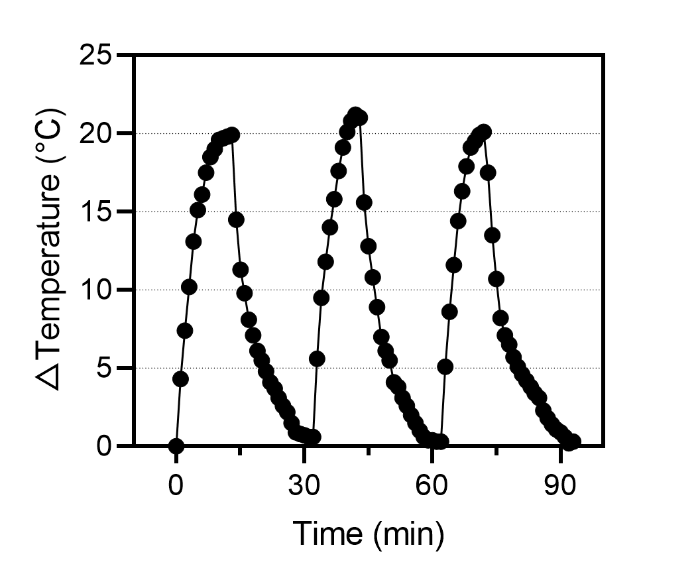


**Figure S1.** Photothermal stability study

**Experimental Procedures**

**Materials:** Polyvinylpyrrolidone (PVP), DOX, gallic acid (GA), and iron dichloride tetrahydrate (FeCl_2_·H_2_O) were purchased from Aladdin Reagent Database Inc (Shanghai, China). All other chemicals and solvents were of analytical or chromatographic grade.

**Synthesis of Fe-GA nanoparticles:**100 mg of Polyvinylpyrrolidone (PVP) was added into 10 mL DI water under sonication at 25 °C. Then, 0.1 mL of FeCl2 aqueous

solution (200 mg mL-1) was mixed with the PVP solution under vigorous stirring. 20 mg of GA was dispersed in 1 mL of water for use. After 30 min of stirring, the FeCl_2_-PVP mixture was mixed with GA solution and stirred overnight. After filtered

with PP microporous membrane (0.22 μm), the resultant was stored in 4 °C refrigerator for the following use.

**Preparation and characterization of FeGA-DOX+AG:** The general protocol for the hydrogel preparation is as follows. The prepared FeGA (100µg in 1mL PBS) and DOX (100µg in 1mL PBS) were mixed into 1% agarose solution to form FeGA-DOX+AG. Scanning electron microscopy (SEM) images were captured on a Hitachi FE-SEM S4800 instrument with an acceleration voltage of 3 kV. UV-vis spectra of samples were recorded by the UV-vis spectrophotometry Lambda 35 (Perkin-Elmer).

**Characterization:** The morphology of FeGA-DOX was observed by transmission electron microscopy (JEM-1200EX, JEOL, Japan). Scanning electron microscope (ZEISS Gemini 300, Zeiss, Germany). The absorption spectra were measured using UV-3600 spectrometer (Shimadzu, Japan).

**Photothermal performance:** The photothermal conversion performance of FeGA-DOX was evaluated. Firstly, FeGA-DOX was dispersed in PBS solution at different concentration (0, 10, 20, 40 μg/ml). Then, these solutions were irradiated by an 808 nm laser with energy density of 1.0 W/cm^2^. At definite time interval, temperature change of these solutions was recorded by an IR camera thermal graphic system (HBT-2A, Hao Bo Technology, China).

**Cell culture:** Osteosarcoma cells (K7M2wt) were cultured in Dulbecco Modifified Eagle’s Medium (DMEM) with 10% fetal bovine serum at 37 °C in a humidified atmosphere with 5 % CO_2_.

**Cell viability assessment:** Osteosarcoma cells (K7M2wt) at a density of 5*10^3^ cells per well were seeded in 96-plates for 24 h. To study cell viability and therapy performance, the culture medium of cells was replaced by 100 μL complete medium with various concentration of FeGA, FeGA-DOX, FeGA+AG, FeGA-DOX+AG (Fe^2+^ concentration of 0, 5, 10, 20, 30, 40 μg/ml) for 24 h. Then wells were added 10 μl cell counting kit 8 reagant and incubated 3 h to assess the absorbance in 450 nm to calculate the cell survival rate. For photothermal therapy assessment *in vitro*, cells were incubated with FeGA+AG or FeGA-DOX+AG and exposed to laser irradiation. After 24h of incubation, cell viability was evaluated by the same method.

**Intracellular ROS Detection:** ROS Assay Kit was used to determined ROS generation in vitro. K7M2wt were seeded in six‐well plates and cultured for 24h. After various treatments, cells were washed by PBS and co-incubated with 20,70-dichlorofluorescein diacetate (DCFH-DA) solution (10 mM) for 30 min, CLSM was used to image and observer the generation of ROS.

**Evaluation of the level of H_2_O_2_ in osteosarcoma cells:** To analysis the production of H_2_O_2_ in different treatment groups, after various treatment, the standard Fluorimetric Hydrogen Peroxide Assay Kit (Sigma-Aldrich, USA) as the fluorescent probe was applied and confocal laser scanning microscope was used to capture the images.

**NADPH oxidase activity measurements:** K7M2wt cells at the density of 1*10^6^ were culture in six-well plates and FeGA-DOX + AG was added in wells and cultured for 24 h. Then the cells were harvested by trypsinization, centrifuged at 25000 rpm for 5 minutes. Next, BCA Protein Assay Kit (Thermo Scientific, Rockford, IL, USA) was used to analyze the protein amount and measure NADPH oxidase activity.

**Animal model:** About 6-week-old female Balb/c nude mice were purchased from Vital River Company (China). The mice were feed under a standard experimental condition. All treatment and for animal were approved by Jilin University Animal Care Facility and National Institutions of Health Guidelines. The right hind leg of each mouse was subcutaneously injected solution which cells (1 × 10^7^) suspended in 100 μL of PBS. All the *in vivo* experiments were completed after the tumour volume reached 200 mm^3^

***In vivo* IR Imaging of mice:** To analyze the change of temperature *in vivo*, saline and FeGA-DOX+AG was intratumoral injected into the K7M2wt tumour-bearing Balb/c mice. Then, the tumour region was irradiated by 808 nm laser with energy density of 1.0 W/cm^2^. IR camera thermal graphic system (HBT-2A, Hao Bo Technology, China) were used to monitored and image the temperature at 0 and 20 minutes.

**Antitumour efficacy：**After the tumour volume reached about 200 mm^3^, all mice were divided into 5 groups (5 mice per group): Group I: saline; Group II: NIR; Group III: FeGA-DOX + AG; Group IV: FeGA + AG + NIR and Group V: FeGA-DOX + AG + NIR, Fe = 6 mg/kg. 200 μL of solutions (saline, FeGA+AG, FeGA-DOX+AG) were injected into the tumour region directly.After 24 h post-injection, the tumour region of mice in Group IV and V were irradiated by an 808 nm laser (1 W/cm^2^, 5 min). Body weight and tumour volume were recorded every 3 days, formula was used to calculated tumour volume: V=ab^2^/2, where a is maximum while b is the minimum diameter in the tumour.

At the end of treatment, the mice were sacrificed. Tumours were collected and fixed with 4% formaldehyde and stored in paraffin for the hematoxylin and eosin (H&E) staining. Terminal deoxynucleotidyl transferase–mediated deoxyuridine triphosphate nick end labeling (TUNEL) and Ki-67 staining analysis was performed on the tumour sections.

**Blood analysis：**To evaluated the toxicity and metabolism of FeGA-DOX in vivo, At the 1st, 7th, and 14th day after injected FeGA-DOX, mice were euthanasia and 1ml blood sample of each mouse was collect for serum biochemistry. These data were compared with the control group which just injected only saline.

**Statistical analysis:** The one-way analysis of variance (ANOVA) followed by the post-Tukey comparison tests were used for statistical analysis. P-value of < 0.05 indicates statistical difference
